# DNA cytosine methylation at the *lexA* promoter of *Escherichia coli* is stationary phase specific

**DOI:** 10.1093/g3journal/jkab409

**Published:** 2021-11-27

**Authors:** Elizabeth B Lewis, Edwin Chen, Matthew J Culyba

**Affiliations:** Division of Infectious Diseases, Department of Medicine, University of Pittsburgh School of Medicine, Pittsburgh, PA 15261, USA

**Keywords:** 5-methylcytosine, DNA methyltransferase, DNA damage response, transcription regulation, LexA, SOS response

## Abstract

The bacterial DNA damage response pathway (SOS response) is composed of a network of genes regulated by a single transcriptional repressor, LexA. The *lexA* promoter, itself, contains two LexA operators, enabling negative feedback. In *Escherichia coli*, the downstream operator contains a conserved DNA cytosine methyltransferase (Dcm) site that is predicted to be methylated to 5-methylcytosine (5mC) specifically during stationary phase growth, suggesting a regulatory role for DNA methylation in the SOS response. To test this, we quantified 5mC at the *lexA* locus, and then examined the effect of LexA on Dcm activity, as well as the impact of this 5mC mark on LexA binding, *lexA* transcription, and SOS response induction. We found that 5mC at the *lexA* promoter is specific to stationary phase growth, but that it does not affect *lexA* expression. Our data support a model where LexA binding at the promoter inhibits Dcm activity without an effect on the SOS regulon.

## Introduction

**Figure 1:**
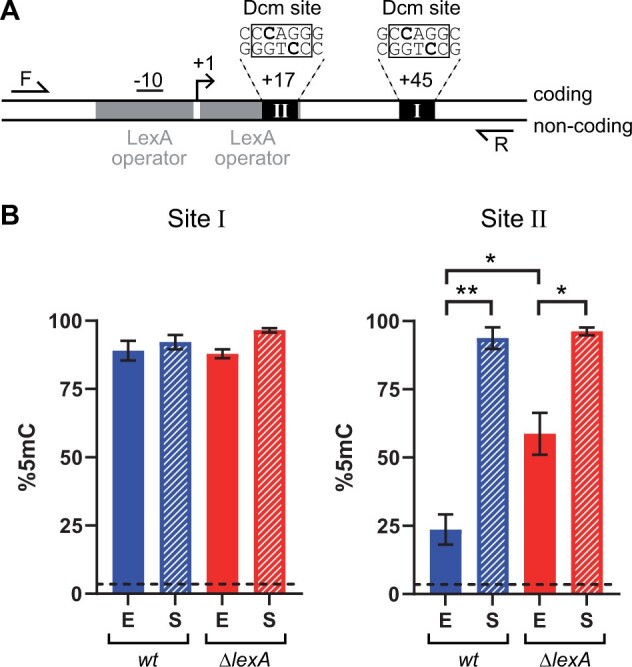
Stationary-phase specific methylation of the *lexA* promoter. (A) Bisulfite sequencing amplicon. Forward (F) and reverse (R) primers capture two Dcm sites (boxed DNA sequences) within the *lexA* promoter region. The internal C residues (bolded) are the targets for 5mC formation. Site I is located at position +45 and does not contain flanking 5′-C residues or overlap with any conserved promoter regions. Site II is located at position +17, contains flanking 5′-C residues, and overlaps with the downstream LexA operator sequence. Both DNA strands are shown (coding, noncoding) and the positions of the transcription start site (arrow), –10 signal sequence, and LexA operators (gray boxes) are indicated. (B) Quantification of 5mC. DNA was harvested from *wt* (blue) or *ΔlexA* (red) cells in either the exponential (E, solid bars) or stationary (S, hashed bars) phase of growth. After bisulfite treatment, strand-specific primers were used to separately amplify and sequence the coding and noncoding strands. Percent 5mC (%5mC) for Site I (left) and Site II (right) was calculated for each strand using the rate of protection from C-to-T conversion. The mean %5mC values for each strand were calculated from independent measurements (*n* = 3–4). Each plotted data point and error bar represents the mean and standard error, respectfully, from combining the mean values of the two stands. Differences in the combined mean values between groups were assessed using a one-way ANOVA with correction for multiple comparisons. Significantly different groups are indicated (^*^P < 0.05, ^**^P < 0.01). Background signal was quantified using DNA harvested from *Δdcm* cells (dashed horizontal line).

The bacterial DNA damage response pathway (SOS response) is composed of a conserved gene network involved in DNA damage repair that enables cells to survive genotoxic insults (Friedberg *et al.* 1995). SOS activation is also mutagenic due to the expression of error-prone polymerases, which hastens bacterial adaptation, including the acquisition of antibiotic resistance (Cirz *et al.* 2005). The pathway is regulated by the LexA and RecA proteins. LexA is a transcriptional repressor and, in *Escherichia coli*, it regulates ∼40 SOS genes. LexA represses transcription by binding to specific 20-bp operator sequences within SOS gene promoters, which prevents RNA polymerase (RNAP) from accessing the promoter. In the setting of DNA damage, RecA becomes activated and stimulates the autoproteolysis of LexA, thereby derepressing SOS genes and turning on the SOS response. The *lexA* promoter, itself, contains two LexA operators, allowing for negative feedback of its expression and more rapid control of the SOS regulon (Brent 1982; Camas *et al.* 2006; Kozuch *et al.* 2020). The downstream LexA operator at the *lexA* promoter of *E. coli* has long been recognized to contain a DNA cytosine methyltransferase (Dcm) site (Figure 1A), which has been postulated to have a regulatory role in the SOS response (Brent 1982), but its significance is untested.

DNA methyltransferases (MTases) catalyze the covalent attachment of a methyl moiety to a specific DNA base ([Bibr jkab409-B18][Bibr jkab409-B18]). In eukaryotes, MTases play an important role in regulating transcription, whereas in prokaryotes, they are most often coexpressed with a restriction enzyme (RE) in the context of a restriction–modification (R–M) system. However, many bacterial MTases do not have a cognate RE and, instead, these “orphan” MTases have regulatory roles. For example, in *Vibrio cholerae*, the 5mC MTase VchM is important for regulating the cell envelope stress response through *rpoE* (σ^E^) (Chao *et al.* 2015). In *E. coli*, DNA adenine MTase regulates DNA replication (Boye and Lobner-Olesen 1990) and mismatch repair (Glickman and Radman 1980) by producing 6-methyladenine at 5′-GATC sites, which modulates the activity of specific DNA-binding proteins. The biological role of Dcm, another orphan MTase found in *E. coli*, is less clear (Marinus and Lobner-Olesen 2014).

Dcm produces 5-methylcytosine (5mC) at the second C at 5′-CCWGG sites. It is conserved in *E. coli* (Militello *et al.* 2012) and other closely related genera of the family *Enterobacteriaceae* (Gomez-Eichelmann *et al.* 1991). Dcm has been implicated in preventing parasitism by the EcoRII R–M system, which also targets 5′-CCWGG sites (Takahashi *et al.* 2002). *E. coli* mutant strains lacking *dcm* exhibit normal growth, but phenotypes that may explain additional biological roles include an association with Tn3 transposition (Yang *et al.* 1989), phage lambda recombination (Korba and Hays 1982), antibiotic resistance (Militello *et al.* 2014), and decreased viability during prolonged stationary phase culture (Militello *et al.* 2020). Dcm has also been implicated in regulating stationary phase gene expression (Kahramanoglou *et al.* 2012; Militello *et al.* 2012). 5mC at Dcm sites is typically considered to be a static epigenetic mark because most Dcm sites are methylated immediately after replication and 5mC is present throughout bacterial growth. However, more recently, a genome-wide analysis of 5mC levels at single base-pair resolution revealed that a subset of Dcm sites exhibit dynamic methylation with respect to growth phase. Sites containing an additional flanking 5′-C, 5′-CCCWGG (underlined), were associated with 5mC specifically during stationary phase growth, and not during exponential phase growth (Kahramanoglou *et al.* 2012). This dynamic methylation raised the possibility that Dcm could play a gene regulatory role at a subset of Dcm sites, although evidence for this remains limited.

It has previously been suggested that 5mC at the Dcm site within the *lexA* promoter could increase *lexA* expression by reducing the LexA binding affinity (Brent 1982), but this mechanism has never been formally tested. Furthermore, if 5mC was a static epigenetic mark, then it could not serve a dynamic regulatory role in the cell. However, we noted that the Dcm site at the *lexA* promoter contains a flanking 5′-C motif on both DNA strands, 5′-CCCAGGG (underlined), suggesting dynamic methylation and, therefore, we hypothesized that Dcm has a role in regulating the SOS response (Kozuch *et al.* 2020). Here, we first use bisulfite sequencing to demonstrate that this Dcm site exhibits stationary phase-specific methylation in *E. coli*. Then, we test the role of this 5mC mark in *lexA* regulation. We show that 5mC does not alter LexA binding affinity, RNAP activity, or *lexA* expression, but rather, LexA inhibits Dcm activity at the promoter. We conclude that stationary phase-specific methylation at the *lexA* promoter does not impact the SOS response.

## Materials and methods

### Bacterial strains and plasmids

All of the strains in this study are derivatives of *E. coli* K12 MG1655. The *ΔlexA* strain has been previously described (Kozuch *et al.* 2020). The *Δdcm* strains (*Δdcm*::*FRT*) were generated by P1 transduction using the *Δdcm*::*FRT*-*kan*-*FRT* strain (JW1944) from the *E. coli* Keio collection (Baba *et al.* 2006) as the donor. The kanamycin resistance cassette was removed by Flp-mediated recombination using plasmid pCP20 and strains were cured of pCP20 by incubation at 42°C, as previously described (Baba *et al.* 2006). The desired gene deletion was confirmed by both PCR and susceptibility to kanamycin.

For *in vivo* transcription rate measurements, the very low copy plasmid pUA66-P*_lexA_gfp* (Ronen *et al.* 2002) was used as previously described (Culyba *et al.* 2018; Kozuch *et al.* 2020). The “AA” derivative construct was created using the Q5 Site-Directed Mutagenesis Kit (New England Biolabs). To facilitate the high DNA yields required for the *in vitro* transcription assays, the *lexA* promoter and downstream *gfp* (P*_lexA_gfp*) construct were amplified from pUA66-P*_lexA_gfp* and cloned into the high copy number pUC19 plasmid (pUC19-P*_lexA_gfp*) using HindIII. Mutations to ablate Dcm sites and remove *gfp* (pUC19-P_*lexA*_*Δgfp*) were introduced into pUC19-P*_lexA_gfp* using the Q5 Site-Directed Mutagenesis Kit (New England Biolabs). All desired mutations were confirmed by DNA sequencing. Primer sequences are given in Supplementary Table S1.

### Bisulfite sequencing

To harvest DNA for bisulfite sequencing, LB broth with inoculated with a 1:100 dilution of an overnight bacterial culture and incubated at 37°C with shaking. Cells were harvested in either exponential phase (absorbance at 595 nm ∼0.3) or stationary phase growth (16 h). For pUC19 derived plasmids, ampicillin was added to the media for plasmid maintenance. The DNeasy Blood and Tissue Kit (Qiagen) was used to harvest genomic DNA and the Plasmid Maxi Kit (Qiagen) was used to harvest plasmid DNA. Bisulfite treatment of the DNA was carried out using the EpiTect Bisulfite Kit (Qiagen) according to the manufacturer’s protocol. After bisulfite treatment, a single PCR reaction from each primer pair was sent for Sanger sequencing. Bisulfite specific primer sequences used for PCR and Sanger sequencing (Supplementary Table S1) were designed using the MethPrimer tool (Li and Dahiya 2002). Site-specific methylation was quantified from Sanger sequencing traces using the Applied Biosystems Variant Analysis module (ThermoFisher). Percent methylation (*%5mC*) was calculated using the formula, $\%5mC\;=\;100\;\times \;\frac{I_{C}}{I_{C}+I_{T}}$, where *I_C_* and *I_T_* represent the intensity values corresponding to the site of interest for C and T, respectively. The number of independent bacterial cultures tested for a given measurement is indicated in the figure caption.

### Electromobility shift assay

Recombinant LexA protein from *E. coli* was overexpressed and purified as previously described (Culyba *et al.* 2018). Protein concentration was determined by the Bradford assay. ^32^P-labeled dsDNA probes were constructed from synthetic DNA oligonucleotides (Integrated DNA Technologies, Coralville, IA, USA) as previously described (Culyba *et al.* 2018). LexA-operator binding reactions were carried out at room temperature in a final volume of 20 µl. Each reaction contained 0.1 nM of ^32^P-labeled probe DNA and the indicated amount of LexA protein. Final reaction conditions were 70 mM Tris–HCl (pH 7.5), 2 mM PIPES, 180 mM NaCl, 10 mM MgCl_2_, 1 µg/ml sonicated salmon sperm DNA, 100 µg/ml bovine serum albumin, 5% glycerol, and 0.006% bromophenol blue. Protein-DNA complexes were separated from free DNA by polyacrylamide gel electrophoresis using 6% polyacrylamide gels cast in 0.5 × Tris-borate-EDTA buffer (TBE). Electrophoresis was carried out in 0.5 × TBE at 4°C at 10 V/cm. DNA bands were quantified by phosphorimaging using the Personal Molecular Imager FX instrument (Bio-Rad) and Quantity One software (Bio-Rad). The fraction of bound probe was plotted as a function of the log of the LexA concentration. The equilibrium constant for the LexA-DNA dissociation reaction (K_d_) and Hill slope parameters were determined by nonlinear regression using the 4-parameter “log(dose) *vs* response” model in Prism (GraphPad). The reported *P*-values are derived from comparison to a model where the parameter is shared between the two datasets.

### *In vitro* transcription rate measurements

The DNA (pUC19-P*_lexA_gfp* or pUC19-P_*lexA*_*Δgfp*) used for *in vitro* transcription assays was prepared from overnight cultures of *E. coli* using either *dcm*^+^ or *Δdcm* cells. Plasmid DNA was linearized using HindIII and the enzyme was heat inactivated at 80°C for 20 min. The molecular beacon (MB) was synthesized using 2′-O-methyl ribonucleotides (Integrated DNA Technologies) (Supplementary Table S1). Transcription reactions were carried out in a final volume of 25.5 µl in a 384-well plate and contained 40 nM (2.4 µg, 1 pmol) plasmid DNA, 0.8 mM of each rNTP (rATP, rCTP, rGTP, and rUTP), and 400 nM MB. Reactions were preheated to 37°C, then initiated by the addition of 0.08 units/µl of RNAP σ70 holoenzyme (New England Biolabs). Final buffer conditions were 30 mM Tris–HCl (pH 7.5), 2 mM PIPES, 120 mM KCl, 30 mM NaCl, 8 mM MgCl_2_, 0.8 mM dithiothreitol, and 0.008% Triton X-100. Fluorescence intensity (FI) was measured every 0.5 min using an Infinite F200 multifunction plate reader (Tecan). Initial rates were estimated by determining the slope of the line from the initial 20 time points (10 min) by linear regression in Prism (GraphPad). The concentration of LexA required to inhibit the reaction rate by 50% (IC_50_) and Hill slope parameters were determined by nonlinear regression using the four-parameter “log(inhibitor) *vs* response” model in Prism (GraphPad). The reported *P*-values are derived from comparison to a model where the parameter is shared between the two datasets. Batch-to-batch variability in RNAP activity was noted, therefore, all replicates and experimental comparisons were made between data derived from the same batch.

### *In vivo* promoter activity measurements

Measurements of promoter activity following a pulse of UV-induced DNA damage and the UV dose–response analysis were performed in live cells using the green fluorescent protein (GFP) reporter plasmid pUA66-P*_lexA_gfp*, or its “AA” derivative, as previously described (Kozuch *et al.* 2020). As described, background fluorescence was determined using a pUA66-P*_lexA_gfp* derivative lacking the *lexA* promoter, but with *gfp* intact, and the background values were subtracted from the experimental values prior to analysis.

### Ultraviolet sensitivity

Overnight cultures were diluted 1000-fold into fresh LB media and streaked in a line onto LB agar plates using a sterile cotton-tipped applicator. The plates were then irradiated with the indicated UV dose using a germicidal lamp (UVP, LLC) set to 254 nm at a distance of 12 in. The fluence rate was determined to be 15 J/s·m^2^ using a UVP UVX radiometer (Analytik Jena). Cardboard was placed over portions of the plate during UV exposure to separate each plate into different UV dose zones. The UV dose of each zone was determined by multiplying the total exposure time by the fluence rate. After irradiation, plates were incubated overnight at 37°C to allow for outgrowth prior to imaging.

## Results

The Dcm site within the *lexA* promoter contains flanking 5′-C residues on both DNA strands, a feature associated with stationary phase-specific methylation (Kahramanoglou *et al.* 2012). To formally test for this dynamic methylation, we quantified 5mC at the *lexA* promoter in *E. coli* during both exponential and stationary phases of growth using bisulfite sequencing. Importantly, we captured two different Dcm sites within the same bisulfite sequencing amplicon (Figure 1A). Dcm Site I served as a control. Its target C is located at position +45 (relative to transcription start site) and does not contain a flanking 5′-C. Dcm Site II is the site of interest. Its target C is located at position +17, overlaps with a LexA operator sequence, and contains flanking 5′-C residues. Sequence alignments of the *lexA* locus demonstrated these features of Site II are conserved in *E. coli* and other closely related *dcm*^+^ species (Supplementary Figure S1). Capture of both sites within a single amplicon allowed for direct comparison of methylation status of the two Dcm sites. We also quantified 5mC separately for each DNA strand (coding and noncoding), but found %5mC values were similar in each case, so averaged the two measurements at each Dcm site (Figure 1B). Using wild-type (*wt*) cells, we found that the control Dcm site exhibited high levels of methylation (89–92% 5mC) in both phases of growth (Figure 1B, “Site I”). In contrast, the site of interest was only partially methylated (24% 5mC) during exponential phase growth and near fully methylated (94% 5mC) in stationary phase (Figure 1B, “Site II”), thus confirming stationary phase-specific methylation at *lexA*.

Dcm Site II overlaps with residues of the LexA operator sequence that are known to make critical protein contacts within the LexA-DNA complex (Zhang *et al.* 2010). Therefore, LexA and Dcm must compete for binding to this DNA at the *lexA* promoter. To understand if LexA interferes with Dcm activity, we also quantified methylation in *ΔlexA* cells. Compared to *wt* cells, we found that methylation was partially restored (59% 5mC) in *ΔlexA* cells during exponential phase growth (Figure 1B, “Site II”). We conclude that LexA contributes to dynamic methylation at Site II. Most likely, this effect is due to LexA competing with Dcm for binding the same DNA site. Although it is possible that deleting *lexA* causes the effect indirectly due to overexpression of the SOS regulon, the *dcm* promoter does not contain LexA operators and *dcm* has not been identified as a LexA regulated gene (Courcelle *et al.* 2001).

**Figure 2:**
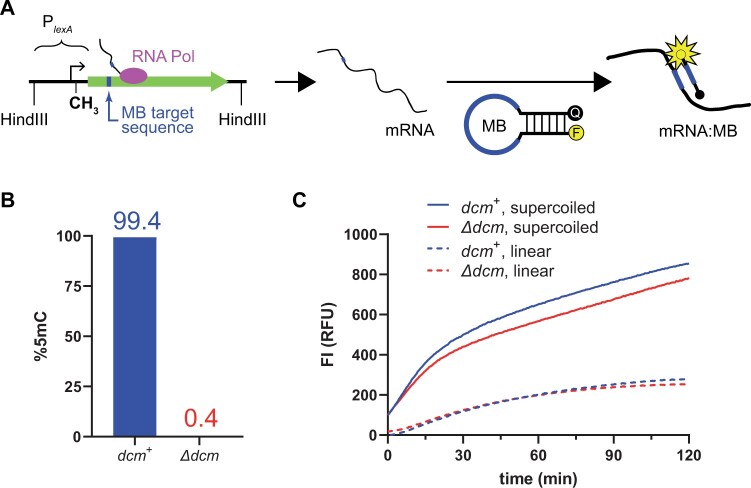
Effect of 5mC on *in vitro* transcription. (A) MB assay. Synthesis of the MB target sequence (blue) is under control of the *lexA* promoter (P_*lexA*_). MB binding to its target mRNA sequence (*gfp*) causes separation of the quencher (black dot, Q) and fluorophore (yellow dot, F) leading to fluorescence. The cloned P*_lexA_gfp* construct can be removed from its plasmid vector by restriction digestion with HindIII. CH_3_ = Dcm Site II. (B) Quantification of 5mC. Bisulfite sequencing was used to quantify 5mC on the noncoding strand of Site II using P*_lexA_gfp* DNA harvested from *dcm*^+^ (blue) or *Δdcm* (red) cells. (C) Transcription kinetics. FI was monitored through time using pUC19-P*_lexA_gfp* DNA harvested from either *dcm*^+^ (blue) or *Δdcm* (red) cells (%5mC quantified in panel B) as the template for RNAP. The DNA was either used directly (supercoiled, solid lines) or was first treated with HindIII (linear, dashed lines). Data points were acquired every 0.5 min and the plotted lines connect the average of two independent replicates at each time point. Background FI was determined using pUC19-P*_lexA_Δgfp* DNA treated in the same manner and plotted data points are corrected for background by subtraction.

Our finding that 5mC at the *lexA* promoter was specific to stationary phase growth raised the possibility that 5mC could regulate *lexA* expression as a function of growth phase. Given the proximity of Site II to the promoter and its overlap with the LexA operator (Figure 1A), we hypothesized that 5mC perturbs *lexA* transcription via two possible mechanisms: Directly altering RNAP activity, or altering LexA repressor activity. To test this, we developed a system to measure the effect of 5mC on RNAP activity at the *lexA* promoter based on a previously described *in vitro* transcription assay that uses a molecular beacon (MB) to detect specific mRNA production (Marras *et al.* 2004). We utilized a construct where *gfp* is under the control of the *lexA* promoter (P*_lexA_gfp*) and employed a previously described MB (Sokolova *et al.* 2013) that targets a specific nucleotide sequence within *gfp* (Figure 2A). By this design, the fluorescence intensity (FI) of the MB is proportional to the concentration of *gfp* mRNA, thus enabling real-time monitoring of RNAP activity at the *lexA* promoter. The P*_lexA_gfp* construct we used contained a total of three Dcm sites, including the site of interest at position +17 (Site II). The other two sites are located at positions –116 and +45, and are not close to any conserved promoter features (Supplementary Figure S1). Although we did not expect 5mC at these distant sites to influence RNAP activity, we introduced a point mutation into each that ablated the Dcm site in order to remove this as a possible confounding variable. To prepare template DNA where Site II was either methylated or nonmethylated, we cloned the P*_lexA_gfp* construct into pUC19 (pUC19-P*_lexA_gfp*) and purified the pUC19-P*_lexA_gfp* plasmid from *dcm*^+^ cells and *Δdcm* cells, respectively. Of note, the pUC19 vector contains five Dcm sites, the closest of which is located at position –320 relative to the *lexA* transcription start site and so is not expected to influence RNAP activity. We used bisulfite sequencing to verify methylation status and, as expected, we found that Site II was not methylated when harvested from *Δdcm* cells (0.4% 5mC) and was fully methylated in *dcm*^+^ cells (99.4% 5mC) (Figure 2B). Transcription reactions were initiated by the addition of purified *E. coli* RNAP σ70 holoenzyme to the methylated and nonmethylated DNA templates and FI was monitored through time (Figure 2C). To ensure MB target specificity, we also generated a construct lacking *gfp* and, as expected, found that the fluorescence signal depended on the presence of *gfp* (Supplementary Figure S2). To facilitate quantitative comparison of transcription from the methylated and nonmethylated templates, we determined initial reaction rates. Of note, plasmid DNA harvested from *E. coli* is negatively supercoiled and we used the supercoiled form for the template DNA in this first set of experiments. We found the rate of *lexA* transcription from *dcm*^+^ cells was 18 RFU/min (95% CI: 13–22) and from *Δdcm* cells was 15 RFU/min (95% CI: 13–17), which was not significantly different (*P* = 0.17).

DNA supercoiling is known to affect RNAP activity (Dorman 2019) and could potentially have confounded our results if *dcm* influenced the supercoiling state of the harvested plasmid DNA. We ruled out this possibility in two ways. First, we directly assessed for differences in supercoiling using agarose gel electrophoresis. We found that plasmid preparations harvested from both *dcm*^+^ and *Δdcm* cells displayed the same migration properties, suggesting no differences in supercoiling (Supplementary Figure S3). Second, we linearized the plasmid DNA using the RE HindIII (Supplementary Figure S3), which cuts out the P*_lexA_gfp* construct from the pUC19 vector (Figure 2A), and then repeated the transcription assay (Figure 2C, “linear”). Linear DNA is in a completely relaxed state, therefore, by subjecting the supercoiled DNA to restriction digestion, it removed any possible differences in supercoiling between the methylated and nonmethylated templates. Additionally, since the restriction digest cuts the P*_lexA_gfp* construct away from the pUC19 vector DNA, the five Dcm sites present in the pUC19 vector can no longer influence RNAP activity in this experiment. Using the linear DNA, we found the rate of *lexA* transcription from *dcm*^+^ cells was 4.4 RFU/min (95% CI: 2.7–6.2) and from *Δdcm* cells was 3.8 RFU/min (95% CI: 1.3–6.3), which was not significantly different (*P* = 0.66). We found that transcription rates were lower on the linear DNA templates as compared to the supercoiled templates, signifying that RNAP activity at the *lexA* promoter is sensitive to supercoiling (Figure 2C). Importantly, however, the linear forms of the methylated and nonmethylated templates still exhibited no difference in transcription rate. These experiments show that 5mC at Site II of the *lexA* promoter has no direct effect on RNAP activity.

**Figure 3:**
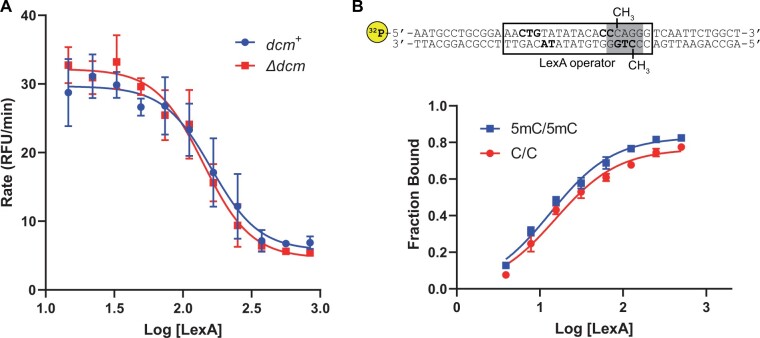
Effect of 5mC on LexA repressor activity. (A) LexA repression of *in vitro* transcription. Transcription rates were determined as in Figure 2 using supercoiled DNA templates and are plotted as a function of [LexA] (nM). Concentrations reflect that of the LexA dimer (=[LexA]/2). Data points and error bars represent the mean and standard error (*n* = 3), respectively, and plotted curves are the result of nonlinear regression. (B) LexA binding affinity. Top: LexA binding was quantified using a ^32^P-labeled dsDNA probe containing the downstream 20 bp LexA operator sequence (boxed) within the *lexA* promoter. Residues known to be important for the LexA-operator interaction are bolded. Target cytosines for 5mC formation (-CH_3_) within the Dcm site (shaded) are indicated. Bottom: The fraction of the probe bound was determined by EMSA and plotted as a function of [LexA] (nM). Concentrations reflect that of the LexA dimer (=[LexA]/2). Data points and error bars represent the mean and standard error (*n* = 2), respectively, and plotted curves are the result of nonlinear regression.

We next tested the effect of 5mC on LexA repressor activity. First, using the *in vitro* transcription assay, we determined the concentration of purified LexA required to inhibit the mRNA synthesis rate by 50% (IC_50_), and then compared the IC_50_ values obtained from the methylated (*dcm*^+^) and nonmethylated (*Δdcm*) templates (Figure 3A). The IC_50_ for the methylated template was 160 nM (95% CI: 100–310) and the IC_50_ for the nonmethylated template was 140 nM (95% CI: 100–200), which were not significantly different (*P* = 0.56). LexA binds to its operator DNA as a dimer (Zhang *et al.* 2010) and there are two operators at the *lexA* promoter (Kozuch *et al.* 2020). To assess for an effect of 5mC on the cooperativity of LexA binding, we also determined the Hill slopes of the repression curves. The Hill slope for the methylated template was –2.6 (95% CI: –7.8 to –0.8) and the Hill slope for the nonmethylated template was –2.4 (95% CI: –5.2 to –1.1), which were not significantly different (*P* = 0.92). This suggested that 5mC does not alter LexA repressor activity at the promoter. Second, to examine the LexA-5mC interaction more specifically, we determined LexA binding affinities to methylated and nonmethylated operator DNA using an electromobility shift assay (EMSA), comparing a LexA operator probe with both target cytosines methylated (5mC/5mC) to the nonmethylated version of the same probe (C/C) (Figure 3B). The K_d_ using the methylated DNA probe (5mC/5mC) was 14 nM (95% CI: 11–17) and the K_d_ using the nonmethylated probe (C/C) was 15 nM (95% CI: 11–21), which were not significantly different (*P* = 0.48). To assess for an effect of 5mC on cooperative binding of the LexA dimer, we also determined the Hill slopes of the binding curves. The Hill slope using the methylated DNA probe (5mC/5mC) was 1.1 (95% CI: 0.9–1.4) and the Hill slope using the nonmethylated probe (C/C) was 1.2 (95% CI: 0.8–1.6), which were not significantly different (*P* = 0.66). Thus, consistent with the results of the transcription assay, we also found no effect of 5mC on LexA binding to its operator. We conclude that 5mC does not alter LexA repressor activity at the promoter.

**Figure 4:**
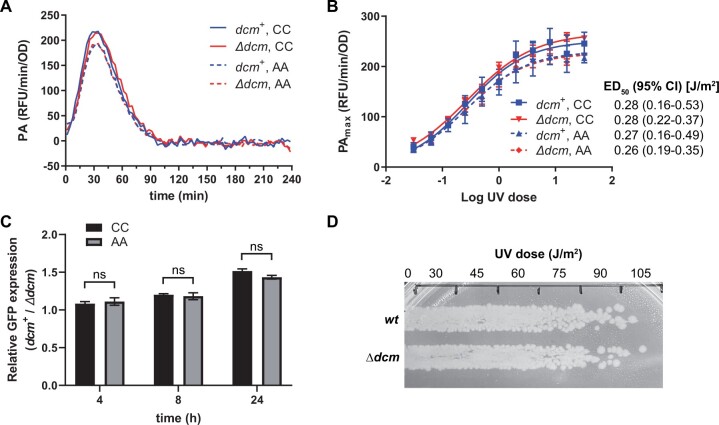
Effect of Dcm on *lexA* promoter activity and UV sensitivity. (A) Promoter activity kinetics. Promoter activity was monitored through time following a UV dose of 2 J/m^2^. Data points were acquired every 0.5 min and the plotted lines connect the average of independent replicates (*n* = 2–4) at each time point. (B) Dose–response analysis. Maximal promoter activity (PA_max_) is plotted as a function of UV dose. Data points and error bars represent the mean and standard error (*n* = 2–4), respectively. Plotted curves and ED_50_ values with associated 95% confidence intervals (95% CI) are the result of nonlinear regression. (C) Relative promoter activity in late stationary phase. GFP FI from the CC and AA promoters was measured at the indicated time points in both *dcm*^+^ and *Δdcm* cells. FI values were normalized to culture density (absorbance at 595 nm) and expressed as the *dcm*^+^/*Δdcm* ratio. Data points and error bars represent the mean and standard error (*n* = 8), respectively. Differences between mean values were assessed by t-tests with correction for multiple comparisons (ns: *P* > 0.05). (D) UV sensitivity. Bacteria were streaked onto LB agar and irradiated with the indicated dose of UV light. Data shown are representative of four independent experiments.

Finally, it remained possible that our *in vitro* experiments did not account for important cellular factors. Therefore, we also measured *lexA* promoter activity in live cells using the P*_lexA_gfp* construct as a GFP reporter. To assess the specific effect of 5mC, we constructed a P*_lexA_gfp* derivative where Dcm Site II was ablated by a 5′-CCCAGG to 5′-AACAGG mutation (underlined), then we compared the “AA” variant to the *wt* “CC”’ variant in both *dcm*^+^ and *Δdcm* cells (Figure 4). This mutation was chosen for two reasons. First, it ablates the Dcm site, but is known to preserve LexA repressor activity based on prior studies where these same LexA operator residues were mutated (Culyba *et al.* 2018; Kozuch *et al.* 2020). Second, in *lexA* promoter alignments of related species, the “AA” promoter variant is conserved amongst more distantly related species, further suggesting that it is functional for LexA repression (Supplementary Figure S1). Comparison of “AA” promoter activity to *wt* “CC” promoter activity in response to a pulse of ultraviolet (UV)-induced DNA damage confirmed that the “AA” promoter was intact and able to be regulated by LexA. As expected, promoter activity increased and then returned to baseline (Figure 4A) in a manner consistent with prior measurements of SOS promoter activity (Ronen *et al.* 2002; Culyba *et al.* 2018; Kozuch *et al.* 2020). Notably, for each promoter, the curves from *dcm*^+^ and *Δdcm* cells were superimposed, indicating no effect on promoter activity by Dcm. Similar results were obtained across a wide range of UV doses and determination of the UV dose required to induce the promoter to 50% maximal activity (ED_50_) also revealed no effect due to Dcm (Figure 4B). We conclude that Dcm does not alter *lexA* promoter activation in response to DNA damage. The above experiments monitored promoter activity through the transition from exponential to stationary phase growth (4 h of culture), but did not capture later times where our bisulfite sequencing confirmed high levels of 5mC (16 h). Therefore, we also measured *gfp* expression at 4, 8, and 24 h of growth. We found that GFP levels were slightly higher for both the “CC” and “AA” promoters in *dcm*^+^ cells (*dcm*^+^/*Δdcm* ratio > 1) across these time points, but that the relative impact of *dcm*^+^ (*dcm*^+^/*Δdcm* ratio) was the same for both the “CC” and “AA” promoters (Figure 4C). This result is consistent with our *in vitro* analyses and demonstrates that there is no effect of 5mC on *lexA* expression. We conclude that 5mC does not affect *lexA* promoter activity. These results predict that loss of Dcm activity would not affect cellular survival during the SOS response to DNA damage. In accordance, we found no difference in UV sensitivity between the *wt* and *Δdcm* strains (Figure 4D).

## Discussion

The presence of a Dcm site within the downstream operator of the *lexA* promoter of *E. coli* has long been speculated to regulate the SOS regulon (Brent 1982), but had not been formally evaluated. We made the additional observation that the Dcm site contained a 5′-C extended motif associated with stationary-phase specific methylation and, therefore, tested the hypothesis that Dcm plays a role in regulating the SOS gene network through methylation of the *lexA* promoter. However, we found no evidence that 5mC had an effect on *lexA* expression, either *in vitro* or *in vivo*. Thus, despite the conservation of this Dcm site in *E. coli* and other closely related *dcm*^+^ species at a location that overlaps a LexA operator (Supplementary Figure S1), we conclude it does not play a role in controlling the SOS regulon through *lexA*. The GFP reporter system used here to study the effect of 5mC *in vivo* is capable of measuring subtle alterations made to SOS promoters and LexA operators (Culyba *et al.* 2018; Kozuch *et al.* 2020). It is, therefore, unlikely that we failed to detect an effect due to a lack of sensitivity. Although it is certainly possible that stationary-phase specific methylation at 5′-C extended Dcm sites could regulate other genes aside from *lexA*, our detailed study of the *lexA* promoter highlights that this must be investigated on a gene-by-gene basis.

Our bisulfite sequencing analysis does conclusively show, however, that the Dcm site within the *lexA* promoter exhibits stationary phase-specific methylation (Figure 1B). In eukaryotes, 5mC can be passively removed by dilution due to new rounds of DNA replication, or alternatively, it can be actively removed by the sequential activities of different cellular enzymes that act to oxidize 5mC, then excise and repair the oxidized base (Kohli and Zhang 2013). In contrast, the removal of 5mC from bacterial cells occurs solely by passive dilution and, therefore, 5mC dynamics are governed by the relative rates of DNA replication and MTase activity. The underlying mechanism that explains our observation of Dcm site-specific hypomethylation during exponential phase must be the result of factor(s) that slow the relative rate of Dcm MTase activity at this particular Dcm site. In this scenario, the high rate of DNA turnover during exponential phase replication outpaces the relatively slow MTase rate at this Dcm site, so that 5mC accumulates only when the rate of DNA replication slows down in stationary phase. There are two main factors that could lower the rate of 5mC formation in a Dcm site-specific manner. First, the 5′-C flanking residue could decrease the rate of MTase target binding or enzymatic activity directly. This mechanism has not been evaluated, but it could explain the association with this sequence motif that was found in the genome-wide study (Kahramanoglou *et al.* 2012). Second, DNA binding proteins could interfere with Dcm activity by preventing its access to the DNA substrate. We found evidence for this latter mechanism at the *lexA* promoter, as the percentage of 5mC was increased in *ΔlexA* cells (Figure 1B). Given that a LexA operator directly overlaps with the Dcm site, this strongly argues that LexA binding prevents Dcm from accessing this site. Notably, 5mC levels were not restored completely in this experiment, so other factors are likely to also play a role. For example, given the Dcm site’s proximity to the transcription start site of *lexA*, it is possible that RNAP also interferes with Dcm activity.

## Data availability

Strains and plasmids are available by request. The authors affirm that all data necessary for confirming the conclusions of the article are present within the article, figures, and tables.

Supplementary material is available at *G3* online.

## Supplementary Material

jkab409_Supplementary_Data
