# Supplementary material for: DNA cytosine methylation at the lexA promoter of Escherichia coli is stationary phase specific
Source: G3 (Bethesda). 2021 Nov 27;12(2):jkab409. doi: 10.1093/g3journal/jkab409 (PMC9210283; doi:10.1093/g3journal/jkab409)
Supplement: jkab409_Supplementary_Data [file jkab409_supplementary_data.pdf]

DNA cytosine methylation at the *lexA* promoter of *Escherichia coli* is stationary phase specific

Elizabeth B. Lewis, Edwin Chen, and Matthew J. Culyba

Supplementary data

Table S1. Oligonucleotides used in this study.

Fig S1. *lexA* promoter alignment.

Fig S2. Molecular beacon target specificity.

Fig S3. Agarose electrophoresis of plasmid preparations.

References for supplementary data

[1] E. Sokolova, E. Spruijt, M.M. Hansen, E. Dubuc, J. Groen, V. Chokkalingam, A. Piruska, H.A. Heus, W.T. Huck, Enhanced transcription rates in membrane-free protocells formed by coacervation of cell lysate, Proc Natl Acad Sci U S A, 110 (2013) 11692-11697.

[2] M.C. Gomez-Eichelmann, A. Levy-Mustri, J. Ramirez-Santos, Presence of 5-methylcytosine in CC(A/T)GG sequences (Dcm methylation) in DNAs from different bacteria, J Bacteriol, 173 (1991) 7692-7694.

[3] R.J. Roberts, T. Vincze, J. Posfai, D. Macelis, REBASE--a database for DNA restriction and modification: enzymes, genes and genomes, Nucleic Acids Res, 38 (2010) D234-236.

NAME/DESCRIPTION	SEQUENCE (5' → 3')

A. ***In vitro*** **transcription**

Molecular beacon	/56-FAM/mCmGmGmC**mAmAmAmUmAmAmAmUmUmUmAmAmGmGmGmUmAmA**mGmCmCmG/3IABkFQ/

B. Mutagenesis

Site I F	TTAACGGCCAGACAACAAGAGGTG
Site I R	CGCTTTCATTCCGCCCCC
Site III F	GCCGTGATTACATGGTGCATTCTGT
Site III R	GACGATAATGGCAATCAGCAC
CC to AA F	TGTATATACAAACAGGGGGCGG
CC to AA R	GTTATGCTGTGAGTATATAC
P_{lexA} *gfp* sequencing	CATCACCATCTAATTCAACAAGAATTG

C. Bisulfite sequencing

gDNA P_{lexA} coding F	GGTTTTGAATATTATGAGTTTTTTGG
gDNA P_{lexA} coding R	TACCTATCTAACTAATATAATCACAA
gDNA P_{lexA} non-coding F	TTGTTTGGTTGATGTGATTA
gDNA P_{lexA} non-coding R	AACTCTAAATACCATAAACTTTCC
P_{lexA} *gfp* non-coding F	GAGGATTTTGGTATATTTG
P_{lexA} *gfp* non-coding R	CACAAAAAATATAAAATCCAC

D. Strain construction

kt	CGGCCACAGTCGATGAATCC
k2	CGGTGCCCTGAATGAACTGC
dcm F	GCTTCTCTATCGCCGTATC
dcm R	TATGTTAACCTGTCGGCCA

E. Electromobility shift assay

lexAop F	AATGCCTGCGGA**AACTGTATATACACCCAGGG**TCAATTCTGGCT
lexAop R	AGCCAGAATTGA**CCCTGGGTGTATATACAGTT**TCCGCAGGCATT
lexAop mC16 F	AATGCCTGCGGA**AACTGTATATACACC/iMe-dC/AGGG**TCAATTCTGGCT
lexAop mC3 R	AGCCAGAATTGA**CC/iMe-dC/TGGGTGTATATACAGTT**TCCGCAGGCATT

Table S1. Oligonucleotides used in this study. Oligonucleotide sequences represent DNA and were purified by desalting, except for the molecular beacon sequence, which was synthesized using 2'-O-methyl RNA bases (indicated by a lowercase 'm' in front of each base) and purified by HPLC (Integrated DNA Technologies). **A. *In vitro* transcription.** The design of the molecular beacon sequence we used to monitor *in vitro* transcription is previously described [1]. Bolded sequence is complementary to *gfp*. /56-FAM/ = 5' FAM (fluorescein) attached with 6 carbon linker; /3IABkFQ/ = 3' Iowa Black quencher. **B. Mutagenesis.** Non-overlapping forward (F) and reverse (R) PCR primer pairs (boxed) were used with the Q5 Site-Directed Mutagenesis Kit (New England Biolabs) to introduce the mutation indicated by the underlined base(s). Desired mutations were confirmed by DNA sequencing using the 'P_{lexA}*gfp* sequencing' primer. **C. Bisulfite sequencing.** Bisulfite treated DNA was amplified using forward (F) and reverse (R) PCR primer pairs (boxed) specific for either the coding or non-coding strand of the *lexA* promoter locus (P_{lexA}) of genomic DNA (gDNA) or the P_{lexA}*gfp* construct. The same two oligonucleotides used to amplify were also used for Sanger sequencing of the amplicon. %5mC was quantified from the oligonucleotide that yielded the highest quality trace. **D. Strain construction.** The listed PCR primers were used in various pairings to either confirm knockout of the *dcm* open reading frame after P1 transduction of the Keio kanamycin cassette, or to confirm cassette removal after induction of the Flp recombinase. The 'kt' and 'k2' primers bind to the cassette and the 'dcm F' and 'dcm R' primers bind to regions of the *dcm* locus flanking the cassette. **E. Electromobility shift assay.** Two oligonucleotides were used to construct each dsDNA probe (boxed). /iMe-dC/ = 5-methyl-2'-deoxycytidine.

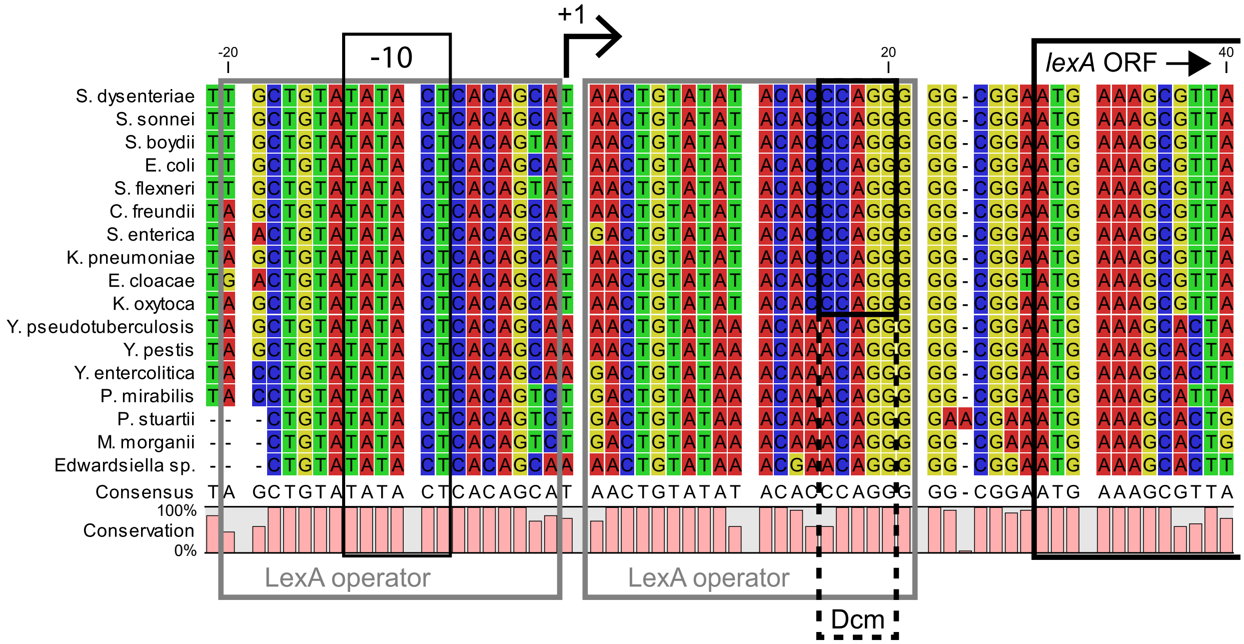

Fig S1. *lexA* promoter alignment. The nucleotide sequence of the *lexA* locus from *E. coli* K12 MG1655 was used as an NCBI BLASTn query sequence, then the consensus sequences of the closest matching species were aligned using CLC Main Workbench software (Qiagen). Residue position numbering is relative to the transcription start site (+1). The -10 RNA polymerase signal sequence, *lexA* open reading frame (ORF), and LexA operators are indicated. An intact Dcm site is indicated by a solid box and its absence is indicated by the dashed box. In addition to *E. coli*, representative strains of *S. sonnei, S. flexneri, C. freundii, S. enterica, K. pneumoniae,* and *K. oxytoca* are known to exhibit 5mC at 5'-CCWGG sites [2]. The REBASE database [3] was also queried for the presence of 5mC Dcm homologues predicted to target 5'-CCWGG. Either confirmed or putative matches were found in genomes from all the species listed in the alignment, except for *Y. pestis*, *Y. pseudotuberculosis*, and *M. morganii.*

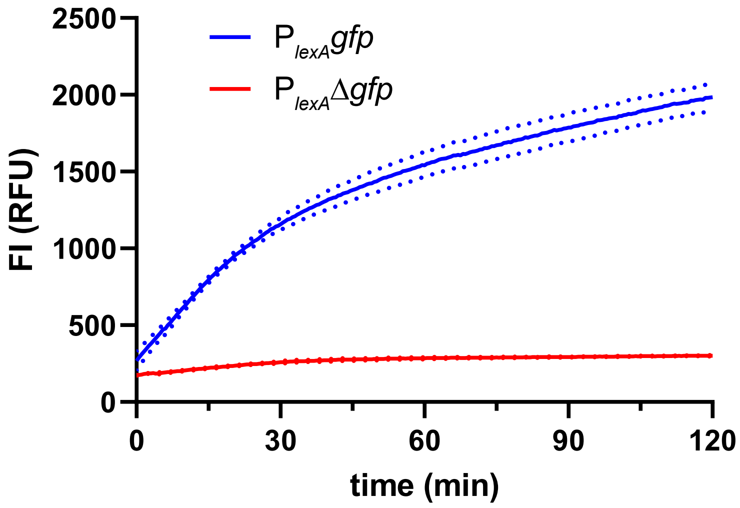

Fig S2. Molecular beacon target specificity. *In vitro* transcription reactions using a DNA template containing the molecular beacon (MB) target sequence (P_{lexA}*gfp*) generated robust signal over time, whereas a DNA template lacking the MB target sequence ($P_{lexA}\Delta$*gfp*) did not. Data points were acquired every 0.5 min. Solid and dotted lines represent the mean and standard error (n=3), respectively.

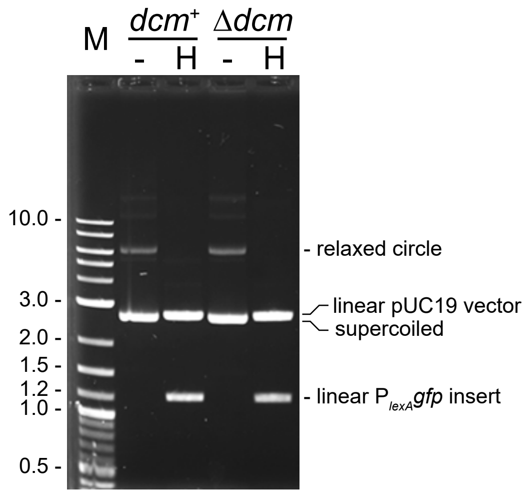

Fig S3. Agarose electrophoresis of plasmid preparations. pUC19-P_{lexA}*gfp* was harvested from *dcm*$^+$ or *Δdcm* cells and either left undigested (-) or digested with HindIII (H). Electrophoresis was carried out in a 0.8% agarose gel with 1x TAE running buffer. After electrophoresis, the gel was stained with ethidium bromide and imaged. The bands corresponding to the supercoiled and relaxed circle forms of the undigested plasmid are indicated. Digestion with HindIII gave the expected two digestion products at ~2.7 kb and ~1.2 kb, which correspond to the linear pUC19 vector and linear P_{lexA}*gfp* insert, respectively.
